# The Yield of Colonoscopy in the Evaluation of Constipation: An Age-Based Analysis of Outcome

**DOI:** 10.3390/jcm13102910

**Published:** 2024-05-15

**Authors:** Fadi Abu Baker, Amir Mari, Randa Taher, Dorin Nicola, Oren Gal, Abdel-Rauf Zeina

**Affiliations:** 1 Department of Gastroenterology and Hepatology, Hillel Yaffe Medical Center, Hadera 38100, Israel; fa_fd@hotmail.com (F.A.B.); randataher12@gmail.com (R.T.); dorin.nicolabathish@gmail.com (D.N.); oren@hymc.gov.il (O.G.); 2 Department of Gastroenterology, Nazareth EMMS Hospital, Nazareth 1613101, Israel; amir.mari@hotmail.com; 3 Department of Radiology, Hillel Yaffe Medical Center, Hadera 38100, Israel

**Keywords:** constipation, colonoscopy, colorectal cancer, inflammatory bowel disease, polyp detection rate

## Abstract

**Background:** Chronic constipation, a prevalent gastrointestinal complaint, exhibits rising incidence and diverse clinical implications, especially among the aging population. This study aims to assess colonoscopy performance in chronic constipation across age groups, comprehensively evaluating diagnostic yield and comparing results with average-risk controls. **Methods:** A retrospective analysis was conducted on 50,578 colonoscopy procedures performed over 12 years, including 5478 constipated patients. An average-risk control group (n = 4100) was included. Data extracted from electronic medical records covered demographics, operational aspects, and colonoscopy findings. The primary outcome measures included the diagnosis rate of colorectal cancer (CRC), polyp detection rate (PDR), and inflammatory bowel disease (IBD) diagnoses in constipated patients versus controls, with age-based and multivariate analyses. **Results:** Constipated patients exhibiting lower rates of adequate bowel preparation (92.7% vs. 85.3%; *p* < 0.001) and a lower cecal intubation rate. No significant variances between CRC and PDR were observed between constipated and controls, except for a potential of a slightly elevated CRC risk in constipated patients older than 80 (2.50% vs. 0% in controls; *p* = 0.07). Multivariate analysis demonstrated, across all age groups, that constipation did not confer an increased risk for CRC or polyp detection. Younger constipated patients exhibited a higher rate of IBD diagnoses (1.7% vs. 0.1% in controls; *p* < 0.001). **Conclusions:** Constipation did not confer an increased risk for CRC or polyps, among any age groups, except for a potential signal of elevated CRC risk in patients older than 80; additionally, it was associated with higher rates of IBD in younger patients.

## 1. Introduction

Constipation represents one of the most prevalent digestive complaints within the general population, thereby inflicting a considerable economic burden [1,2,3]. The prevalence of chronic constipation escalates with advancing age, manifesting a particularly dramatic increase in patients aged 65 years or older [4]. Symptoms of constipation encompass infrequent bowel movements, strenuous defecation, and discomfort. Nevertheless, it is noteworthy to acknowledge that disparate definitions of constipation have been employed in various studies, prompting the adoption of more comprehensive criteria for diagnosing chronic functional constipation, as defined by the Rome IV diagnostic criteria elsewhere [5].

The initial assessment of patients presenting with chronic constipation necessitates a meticulous evaluation, including a comprehensive medical history, physical examination, and laboratory investigations to rule out secondary causes. Endoscopic and radiological studies should be reserved for select individuals only [6,7].

Notably, the relationship between constipation and colorectal cancer (CRC) has garnered substantial research attention. A prevailing hypothesis posits that delayed colonic transit may constitute an etiological mechanism in the development of CRC, owing to the extended exposure of the large bowel mucosa to carcinogenic agents in the stool, thereby culminating in an augmented risk of CRC [8,9]. Nevertheless, it remains unclear whether constipation itself is directly associated with the development of adenomas or CRC. Studies investigating the relationship between chronic constipation and colorectal cancer have produced predominantly negative and inconclusive results [10,11,12]. Consequently, some clinical guidelines advise against performing gastrointestinal endoscopy as part of the initial assessment for patients presenting with chronic constipation unless alarm features or suspicions of organic gastrointestinal disease exist. However, patients aged 50 years or older who have not undergone prior colorectal cancer screening should undergo screening [13].

Indeed, a limited number of studies have explored the diagnostic yield of colonoscopy when employed solely for constipation as an indication, particularly across different age groups. These studies have collectively revealed a consistently low incidence of pathological findings [14,15]. The present study endeavors to ascertain the prevalence of colonic neoplasia and other pathologies uncovered through diagnostic colonoscopy in patients presenting with constipation as their exclusive symptom. Additionally, it aims to perform an age-based analysis of outcomes and compare the findings with an “average-risk” control group.

## 2. Methods

This study was designed as a retrospective analysis, featuring a substantial cohort of consecutive patients who underwent colonoscopy procedures over a 12-year span (2008–2020) at the gastroenterology department of Hillel Yaffe Medical Center, a university-affiliated hospital in Israel. All eligible patients who underwent diagnostic colonoscopy solely for constipation were included. Demographic, clinical, and endoscopic data were extracted from the electronic medical records. Exclusion criteria encompassed patients under 18 years, those with concomitant indications for endoscopic investigations or alarm symptoms, a personal history of CRC, prior colectomy, or those with incomplete relevant data sets. Simultaneously, we included an “average-risk” control group, consisting of patients referred for their first screening colonoscopies during the same period. Data collected from medical records included age, gender, ethnicity, family history of CRC, preparation regimen and quality (categorized as adequate or inadequate based on the Aronchick Scale), the extent of colonoscopy, sedative doses administered during the procedure, and colonoscopy findings. Colonoscopy findings encompassed the detection rate of polyps, the rate of CRC diagnoses, the presence of diverticula, melanosis coli, internal and external hemorrhoids, and colitis. In cases where endoscopic findings suggested CRC, histopathology reports were reviewed to confirm the diagnosis. The primary outcome measure was the prevalence of CRC and polyps in constipated patients, juxtaposed with the average-risk controls. Furthermore, an age-based analysis of outcomes was conducted to explore CRC and polyp diagnosis rates within each age group, with a multivariate analysis was employed to identify predictors of an elevated polyp detection rate and CRC.

### Statistical Analysis

Descriptive statistics, including means, standard deviations, and percentages, were used to summarize all study parameters. Differences between the constipated patient group and the average-risk control group were assessed using Fisher’s exact test for categorical parameters and the *t*-test for quantitative parameters. Multivariate logistic regression analysis was employed to determine the effect of independent variables associated with CRC and polyp detection rates, quantified as odds ratios with 95% confidence intervals (95% CI). Significance was denoted by a threshold of *p* < 0.05. All statistical analyses were conducted using SPSS version 25.

## 3. Results

We conducted an extensive review of 50,578 colonoscopy procedures performed during the study period. Overall, the control group encompassed individuals a total of 4100 patients aged 50 or above, [range: 50–89]. The constipation cohort included 5478 patients and was further divided into two distinct groups: 1119 patients aged below 50 years [range: 16–49] comprised the younger group, and 4359 patients aged 50 years or above [range: 50–96] comprised the older constipation group (Table 1).

Expectedly, variations in ages were observed among the studied groups. A statistically significant difference in gender distribution was also noted among the groups. Among control patients, a slight male predominance (54%) was observed, whereas a lower proportion of males among younger (40%) and older (48%) constipated patients was documented.

In all studied groups, the majority of patients were of Jewish ethnicity, reflecting the baseline representation of this demographic in the population. However, this prominence was particularly evident in the control group, with 95% of control patients being of Jewish affiliation.

The majority of procedures for both groups were performed in outpatient settings, particularly for controls. The vast majority of patients in both study groups adhered to a polyethylene glycol (PEG)-based bowel preparation regimen, predominantly utilizing Moviprep © (ingredients: macrogol 3350, sodium sulfate anhydrous, sodium chloride, potassium chloride, ascorbic acid, and sodium ascorbate) at rates of 86.0% for younger constipated patients, 84.4% for older constipated patients, and 85.6% for controls (*p*^1,2^ > 0.05). Meroken © (ingredients: polyethylene glycol 3350, sodium chloride, sodium bicarbonate, potassium chloride) was used by 9.3% of younger constipated patients, 10.1% of older constipated patients, and 8.8% of controls (*p*^1,2^ > 0.05), while the remaining percentage received other/unspecified preparations”.

Additionally, all patients received sedation during colonoscopy procedures, predominantly from non-anesthesiologists aiming for moderate sedation. Anesthesiologist involvement was notably higher among constipated patients, with 3.1% of younger constipated patients, 2.8% of older constipated patients, and 1.7% of control patients requiring their services (*p*^1,2^ < 0.001). Among patients sedated by non-anesthesiologists, a combination of BDZ and intermittent bolus administration with propofol was utilized to achieve moderate sedation, with no significant differences observed between the study groups (94.6% of younger constipated patients vs. 93.3% of older constipated patients and 93.7% of controls; *p*^1,2^ > 0.05). Conversely, the remaining patients were sedated by propofol alone, with no statistically significant differences observed between the groups (*p*^1,2^ > 0.05). Notably, constipated patients exhibited a higher need for high-dose propofol sedation, with 6% of younger constipated patients, 7% of older constipated patients, and 3% of control patients requiring high-dose sedation (*p*^1,2^ < 0.001).

Moreover, constipated patients exhibited distinct patterns in endoscopic findings compared to the control group. Notably, they exhibited a lower rate of achieving adequate bowel preparation, (92.7% vs. 85.3%; *p* < 0.001) and (92.7% vs. 87.3%; *p* < 0.0001), in the controls vs. younger and older constipation groups, respectively. Furthermore, constipated patients displayed a significantly lower rate of cecal intubation (96.1% vs. (85% and 84%); *p* < 0.001).

Regarding the detection of polyps, constipated patients, especially in the younger group, had a significantly lower polyp detection rate compared to the control group (6.8% vs. 21.5%; *p* < 0.01). Conversely, constipated patients aged 50–96 showed a similar polyp detection rate to controls (20.4% vs. 21.5%; *p* = 0.15). The colorectal cancer detection rate was low for all groups, with non-significant differences between younger constipated patients and controls, but was significantly elevated in older constipated patients compared to controls (0.6% vs. 0.3%; *p* = 0.03). Younger constipated patients had a notably higher rate of IBD compared to control patients (1.7% vs. 0.1%; *p*^1,2^ < 0.001). This was also evident among older constipated patients but to lower extent (0.5% vs. 0.1%; *p*^1,2^ < 0.001).

**Figure 1:**
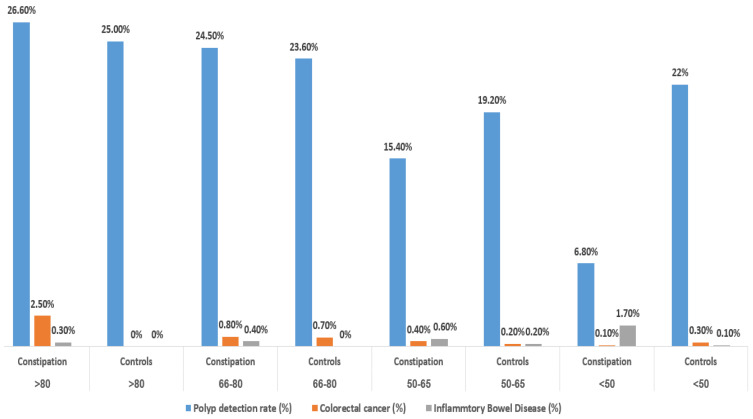
Primary Outcomes by Age-Group: Constipated Patients vs. Controls.

In age-group sub analysis, PDR, CRC, and IBD diagnosis rates varied across age groups (Figure 1), PDRs were comparable across all age groups between constipated patients and control with mild non-significant variations, except in the younger age group (below 50 years), where constipated patients displayed a PDR of 6.80%, significantly lower than control patients, who had a PDR of 22% (*p* < 0.001).

Similarly, no significant variances in CRC diagnosis rates where noted among age groups, except for patients older than 80 years, where the CRC rate was significantly higher compared to younger age groups (2.5%); however, when compared to controls (2.5% vs. 0.0%), this was only close to significance (*p* = 0.07), possibly due to the low number of patients in this age category (12 patients in controls and 420 in constipated patients). In terms of IBD diagnoses rates, these where higher in constipated patients across all age-categories and were particularly and significantly higher in younger constipated patients (below 50 years) (1.70%, vs. 0.10%; *p* < 0.01).

Finally, in overall and age-category specific multivariate analysis (Table 2), only age was predictor of increased PDR and CRC diagnosis rate (OR = 1.034, 95% CI 1.03–1.04; *p* < 0.01) and (OR= 1.04, 95% CI 1.02–1.07; *p* < 0.01), respectively. In contrast, inadequate bowel preparation (OR = 1.192, 95% CI 1.07–1.33; *p* = 0.02) was predictor of lower PDR diagnosis rates. Constipation did not confer increased risk for polyp detection (OR = 1.091, 95% CI 0.84–1.41; *p* = 0.51) or CRC diagnosis (OR = 0.62, 95% CI 0.32–1.18; *p* = 0.15).

## 4. Discussion

According to the current study findings, a significant portion of endoscopic procedures in our clinical practice, approximately 15–18%, are performed to investigate constipation as the primary presentation, with a substantial proportion, approximately 20–25%, being performed in younger patients below the age of 50. This observation highlights the importance of addressing various aspects of the endoscopic evaluation of constipation and necessitates the inclusion of average-risk patients as controls for meaningful comparisons.

Our study revealed several critical findings, the first of which is the substantial difference in bowel preparation quality between constipated patients and controls. In accordance with previous studies [16,17], we observed that constipated patients, including younger patients with constipation, exhibited a lower rate of adequate bowel preparation (92.7% vs. 85.3%; *p* < 0.001) compared to controls. This suboptimal bowel preparation, a recognized challenge in the evaluation of constipation, not only impacts the quality of colonoscopy but also results in a decreased cecal intubation rate (96.1% vs. 85% and 84%; *p* < 0.001). Given these findings, it is imperative to emphasize the critical importance of raising awareness among both patients and healthcare providers, highlighting the significance of strict adherence to bowel preparation regimens. Furthermore, a tailored and enhanced approach to bowel preparation may offer significant potential for improving overall effectiveness in this context [18].

Moreover, we observed an increased need for higher doses of propofol sedation and anesthesiologist involvement among constipated patients. This might be attributed to the potentially longer duration of colonoscopy procedures in this patient group, possibly due to the challenges posed by poor bowel preparation. Although we lacked data on the exact duration of the procedures, this finding suggests that addressing the issue of bowel preparation is essential to optimize the efficiency of colonoscopy in constipated patients. It is noteworthy to mention that, to the best of our knowledge, data regarding sedation requirements specific to constipated patients have not been directly reported in the existing literature.

One of the major findings in our study is the lack of a significantly increased risk of colorectal cancer (CRC) or polyp detection rate (PDR) in constipated patients across all age groups. This observation is consistent with recent evidence and adds to the growing body of literature suggesting that chronic constipation may not be directly associated with an elevated risk of CRC or adenoma development [12,19]. While some previous studies have indicated a potential link between chronic constipation and colon cancer [20,21], a comprehensive meta-analysis did not find a significant association between these factors [22]. These results collectively emphasize the importance of not routinely subjecting constipated patients to endoscopic evaluations for CRC unless alarm features or other indications are present [23].

Although the overall rate of CRC was low in our study, a possible signal of an increased risk of CRC was observed in older constipated patients, particularly in those aged 80 and older. However, this potential association was not firmly established, and the limited number of controls in this age category emphasizes the need for further investigation in a larger cohort.

An interesting finding in our study was the significantly higher rate of inflammatory bowel disease (IBD) diagnoses in constipated patients, particularly in the younger age group (1.7% vs. 0.1%; *p* < 0.001). This observation suggests that constipation in younger patients should raise the possibility of underlying IBD, an aspect that has not been extensively covered in the existing literature [24]. It underscores the importance of thorough clinical and laboratory evaluations to assess for potential IBD in these patients.

Our study has several limitations that should be acknowledged. Firstly, the retrospective nature of the study design may have introduced inherent biases and limitations in data collection. Although we meticulously extracted data from electronic medical records, the potential for missing data or incomplete documentation remains a concern. Additionally, due to the single-center nature of the study, the fact that our findings may not encompass all possible factors contributing to constipation, and that the specific etiology and characteristics of constipation in our patient population may differ from those in other regions or healthcare systems, the generalizability of our results should be interpreted within the context of the specific patient population and clinical practices studied.

In conclusion, constipated patients, compared to average-risk patients, had a high rate of poor bowel preparation and incomplete exams. However, constipation does not significantly increase the risk of CRC or polyps, across all age groups, except for a potential signal in older patients aged 80 and above. Notably, younger constipated patients exhibit a higher rate of IBD diagnoses.

## Figures and Tables

**Table 1:** Baseline characteristics of both constipation groups and controls.

Group	Constipation; Age ≥ 50; [50–96]	Constipation; Age > 50; [16–49]	Control; Age ≥ 50; [50–89]	*p* Value
	n = 4359	n = 1119	n = 4100	
Demographics				
Age (years)	10.2 ± 66.7	9.2 ± 39.1	6.8 ± 59.4	***p*^1,2^ < 0.001**
Sex (Male)	2074 (48%)	446 (40%)	2206 (54%)	***p*^1,2^ < 0.001**
Ethnicity (Jewish)	3754 (86%)	764 (68%)	3889 (95%)	***p*^1,2^ < 0.001**
Endoscopy—Setting and Sedation				
Setting (Outpatient)	3778 (87%)	1033 (92.3%)	4019 (98%)	***p*^1,2^ < 0.001**
Sedation provider (AN)	116 (2.8%)	35 (3.1%)	71 (1.7%)	***p*^1,2^ < 0.001**
Sedation provider (NAN)				
Propofol monotherapy	229 (5.4%)	73 (6.7%)	267 (6.6%)	*p*^1,2^ > 0.05
Propfol + BDZ	4014 (94.6%)	1011 (93.3%)	3762 (93.7%)	*p*^1,2^ > 0.05
PS-High dose	307 (7%)	70 (6%)	126 (3%)	***p*^1,2^ < 0.001**
Endoscopic Findings				
Bowel preparation				
Moviprep ©	3679 (84.4%)	964 (86.0%)	3505 (85.6%)	*p*^1,2^ > 0.05
Meroken ©	470 (10.1%)	104 (9.3%)	361 (8.8%)	
Other/Unspecified	210 (4.8%)	51 (4.6%)	234 (5.7%)	
Bowel preparation	3720 (85.3%)	978 (87.3%)	5024 (92.7%)	***p*^1,2^ < 0.001**
(Adequate)				
Cecal intubation	3640 (84.0%)	951 (85%)	3945 (96.1%)	***p*^1,2^ < 0.001**
Terminal ileum intubation	60 (1.4%)	29 (2.4%)	64 (1.6%)	*p*^1,2^ > 0.05
Melanosis coli	139 (3.2%)	27 (2.4%)	1 (0.0%)	***p*^1,2^ < 0.001**
Polyp detection rate	890 (20.4%)	76 (6.8%)	880 (21.5%)	***p***^1^ = 0.65
				***p*^2^ < 0.01**
Colorectal cancer	27 (0.6%)	1 (0.1%)	12 (0.3%)	***p*^1^ = 0.03**
				*p*^2^ = 0.15
Inflammatory bowel disease	21 (0.5%)	19 (1.7%)	6 (0.1%)	***p*^1,2^ < 0.001**

Abbreviations: AN: anesthesiologist, BDZ: benzodiazepine, NAN: non-anesthesiologist, PS: propofol sedation.

**Table 2:** Predictors of increased colorectal cancer and polyp detection rates. A multivariate analysis.

Parameter	*p* Value	Odds Ratio	Confidence Interval
Polyp detection predictors			
Patient’s age (years)	<0.001	1.034	1.03–1.04
Sex (male)	<0.001	1.514	1.36–1.67
Ethnicity (Jewish)	0.07	1.171	0.98–1.39
Constipation	0.51	1.091	0.84–1.41
Bowel preparation (adequate)	0.002	1.192	1.07–1.33
Inflammatory bowel disease	0.046	0.226	0.05–0.94
Colorectal cancer predictors			
Patient’s age (years)	<0.01	1.044	1.02–1.07
Sex (male)	0.81	0.936	0.54–1.62
Ethnicity (Jewish)	0.92	0.958	0.40–2.29
Constipation	0.15	0.620	0.32–1.18
Bowel preparation (adequate)	0.73	1.108	0.61–1.99
Inflammatory bowel disease	0.08	1.161	0.96–1.33

## Data Availability

The data presented in this study are available on request from the corresponding author due to restrictions imposed by the local committee and hospital policies regarding patient privacy and confidentiality.
